# Impact of Radiochemotherapy on Immune Cell Subtypes in High-Grade Glioma Patients

**DOI:** 10.3389/fonc.2020.00089

**Published:** 2020-02-14

**Authors:** Valérie Dutoit, Géraldine Philippin, Valérie Widmer, Eliana Marinari, Aurélie Vuilleumier, Denis Migliorini, Karl Schaller, Pierre-Yves Dietrich

**Affiliations:** ^1^Laboratory of Tumor Immunology and Center of Oncology, Geneva University Hospital, Geneva, Switzerland; ^2^Translational Research Center for Oncohematology, Department of Internal Medicine Specialties, University of Geneva, Geneva, Switzerland; ^3^Department of Oncology, Geneva University Hospital, Geneva, Switzerland; ^4^Department of Clinical Neurosciences, Division of Neurosurgery, Geneva University Hospital, Geneva, Switzerland

**Keywords:** glioma, temozolomide, radiotherapy, immunotherapy, lymphopenia, cancer vaccines, immune subsets

## Abstract

Glioblastoma is a dreadful disease with very poor prognosis, median overall survival being <2 years despite standard-of-care treatment. This has led to the development of alternative strategies, among which immunotherapy is being actively tested. In particular, many clinical trials of therapeutic vaccination using peptides or tumor cells are ongoing. A major issue in implementing therapeutic vaccines in patients with high-grade glioma is that immune responses have to be elicited in the context of immunosuppressive treatments. Indeed, radiotherapy, chemotherapy, and steroids, which are part of the standard of care for patients with glioblastoma, are known to deplete leukocytes. Whether lymphopenia is beneficial or detrimental to elicitation of efficient immune responses is still debated. Here, in order to determine the impact of standard radiochemotherapy on immune cell subsets, we analyzed the phenotype and function of immune populations in 25 patients with high-grade glioma along concomitant radiochemotherapy and adjuvant chemotherapy with temozolomide. Thirteen healthy individuals were studied along the same period. We show that absolute T and B cell counts are reduced upon concomitant radiochemotherapy. Importantly, T cell counts were not restored long-term after discontinuation of treatment. In addition, the percentage of T regulatory cells among CD4 T cells was increased during the same period and was not decreased upon treatment discontinuation. Finally, we show that the ability of T cells to proliferate is transiently reduced after concomitant radiochemotherapy but is restored at the time of adjuvant TMZ cycles. Although not experimentally validated, transient reduction in proliferation associated with strong lymphopenia during radiochemotherapy may suggest that vaccine-induced T cell stimulation would be suboptimal in that period and that therapeutic vaccination should be performed outside radiochemotherapy administration. In addition, strategies aiming at depleting Treg cells should be implemented in future trials.

## Introduction

OMS grade IV glioma (glioblastoma, GBM) is the most aggressive form of brain tumors, and is a main cause of death by cancer in children and young adults. Despite state-of-the art treatments, the prognosis of GBM remains dismal, with a median overall survival of <2 years (1, 2). This poor prognosis has prompted the development of novel therapeutic options, among which immunotherapy is showing great promise (3).

Standard of care treatment for newly diagnosed adult patients suffering from primary GBM is maximal safe surgical resection followed by 6 weeks of radiochemotherapy consisting of 60 Gy radiotherapy administrated in 30 fractions and temozolomide (TMZ) 75 mg/m^2^ daily, completed by 6 additional cycles of TMZ (150–200 mg/m^2^, 5 days per month) (4). A major prognosis factor for patients receiving TMZ is the methylation status of the O6-methylguanine DNA methyltransferase (MGMT) gene promoter region, with patients having a methylated MGMT displaying improved survival (5). IDH1/2 mutation is another positive prognosis factor and is associated mostly with GBM arising from lower grade tumors (secondary GBM) (6). The only modality recently made available for the treatment of newly diagnosed GBM is the use of tumor-treating fields, however with debated efficacy (4, 7). Treatment of recurrent GBM includes surgery, irradiation, and chemotherapy with nitrosoureas or bevacizumab, although the efficacy of these treatments is limited (4).

Immunotherapy is now considered a promising option for glioma and numerous trials are ongoing (3, 8, 9). These include therapeutic vaccines (10), T cell therapy with chimeric antigen receptors (11), and immune checkpoint inhibitors (12). However, efficacy of strategies aiming at stimulating the immune system in the context of standard of care in patients with GBM is still an open question. Radiochemotherapy induces a profound immunosuppression, which adds to that induced by the disease itself and by the use of steroids, perioperatively, or during treatment (13), suggesting that immunotherapies might not be efficient in that context. On the other hand, chemotherapy-induced immunosuppression has been shown to induce IL-2, IL-7, and IL-15 and mediate immune reconstitution (14), potentially being beneficial for the elicitation of antitumor immune responses. Studies in mice (15, 16), and humans (17, 18) showed that myeloablative TMZ allows the elicitation of high magnitude vaccine-specific immune responses and chimeric antigen receptor T cell persistence (19), whereas other reports suggest that GBM-associated lymphopenia might prevent elicitation of efficient immune responses (20–23). Of particular interest is the potential of T regulatory (Treg) cell depletion by TMZ or radiotherapy; however, reports showed that current TMZ dosing was not able to deplete Tregs (20, 24, 25). Radiation-related lymphopenia has been recognized for many years (26) and it was shown in high-grade glioma patients treated before the era of TMZ that radiotherapy induces grade 4 lymphopenia in a significant fraction of patients (27), even though the brain only was targeted (28). Overall, grade 3–4 lymphopenia was shown to occur in the majority of patients receiving standard-of-care treatment, with severe lymphopenia being an independent predictor of poor survival (29), and immune cell modulation was shown to be mostly restricted to CD4 T cells and B lymphocytes (21, 26). Finally, the relative contribution of each of the steroids, radiotherapy, and chemotherapy in treatment-induced patient lymphopenia is yet to be understood.

The recognition of treatment-associated lymphopenia has questioned the use of immunotherapeutic strategies in cancer patients. Whereas it was first thought to be an obstacle to induction of anti-tumor immune responses, it is now accepted that radiotherapy and chemotherapy can synergize with immunotherapy (30, 31). Radiotherapy has been associated with the abscopal effect, namely the regression in sporadic cases of distant non-irradiated tumors (32), which has been suggested to be mediated by immune activation after radiotherapy-induced immunogenic tumor cell death. Radiotherapy is also known to upregulate MHC class I molecules and improve antigen expression (31). Similarly, it has been shown that low-dose cytotoxic agents induce better immune response by increasing antigen presentation on dendritic cells, through immunogenic tumor cell death and enhanced visibility of tumor cells to T cells (30). However, these effects are not observed at the high doses at which chemotherapeutic drugs are currently used, casting doubts on the feasibility of these combinations. Here, in order to determine the impact of the standard of care treatment on the immune system of patients with GBM, we prospectively analyzed the number, phenotype, and function of different immune cell populations along time. Long-term follow-up was performed in a fraction of patients.

## Materials and Methods

### Patients

**Figure 1:**
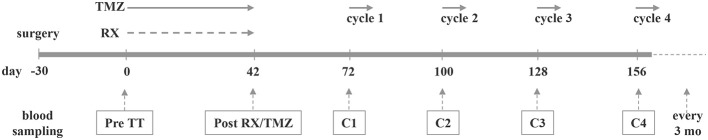
Study scheme. Patients underwent surgery (day −30) followed by concomitant radiotherapy (60 Gy) and TMZ (75 mg/m^2^) (day 0–42). After a 4-week break (day 42–72), patients were given adjuvant TMZ (150–200 mg/m^2^) at day 1–5 of each month. TMZ was discontinued in case of progression or intolerability. Time points for peripheral blood analysis are indicated in boxes (pre TT: pre RX/TMZ therapy, post-RX/TMZ: post-RX/TMZ therapy, C1-4, at day 1 of TMZ cycle 1–4).

Between June 2009 and September 2012, 24 patients with GBM and one patient with grade III astrocytoma were enrolled in this study. This study was carried out in accordance with the recommendations of the Swiss Ethics Committees on research involving humans, with written informed consent from all subjects. All subjects gave written informed consent in accordance with the Declaration of Helsinki. The protocol was approved by the Ethics Committee of Geneva, Switzerland. Adult patients were included if they were scheduled to receive standard radiochemotherapy. Thirteen healthy individuals who were spouses of the patients were included as controls and similarly gave written informed consent. Patients underwent standard of care treatment for GBM (Figure 1) including concomitant TMZ (75 mg/m^2^) and radiotherapy (RX, 60 Gy) followed by cycles of adjuvant TMZ (150–200 mg/m^2^) (1). The duration of concomitant TMZ/RX administration varied from 34 to 49 days (mean 42 days). The dose of TMZ received during this period was 4,250–7,050 mg (mean 5,580 mg, Table 1). Peripheral blood samples (30 ml) were collected at various time points as depicted in Figure 1: pre TT (before RX/TMZ initiation), post-RX/TMZ (after the 6-wk course of concomitant RX/TMZ administration), C1, C2, C3, and C4 (at the beginning of cycles 1–4 of adjuvant TMZ administration). When possible, samples were collected every 3 months thereafter. Lymphopenia was defined as grade 1: <1,000 cells/μl, grade 2: <800 cells/μl, grade 3: <500 cells/μl, grade 4 <200 cells/μl. Neutropenia was defined as <1,000 cells/μl. IDH1/2 mutational status and MGMT promoter methylation status were unknown for the majority of patients, as recruitment occurred in 2009–2012, before systematic analysis of these parameters (Table 1).

**Table 1:** Patients demographics and characteristics.

**Patient characteristics**	**All patients (*n* = 25)**	
Age: median (range)	53.5 (25–72)	
Gender: *n* males (%)	12 (48)	
Pathology		
GBM (*n*)	24 (96)	
Grade III astrocytoma (*n*)	1 (4)	
ECOG status		
0: *n* (%)	17 (68)	
1: *n* (%)	8 (32)	
MGMT promoter methylation status		
Methylated: *n* (%)	7 (28)	
Unmethylated: *n* (%)	4 (16)	
Unknown: *n* (%)	13 (56)	
IDH1/2 mutational status		
Wild type: *n* (%)	5 (20)	
Mutated: *n* (%)	1 (4)	
Unknown: *n* (%)	19 (76)	
TMZ dose (mg) received during concomitant RX/TMZ treatment: mean (range)	5,580 (4,250–7,050)	
Radiotherapy dose (Gy) received during concomitant RX/TMZ treatment: mean (range)	60.4 (59.4–72)	
Cycles adjuvant TMZ: mean (range)	5.5 (0–12)	
TMZ dose (mg) received during adjuvant TMZ treatment: mean (range)	7,385 (0–12,550)	
Further treatments		
Bevacizumab: *n* (%)	19 (76)	
Irinotecan: *n* (%)	2 (8)	
Lomustine: *n* (%)	4 (16)	
TMZ: *n* (%)	2 (8)	
None: *n* (%)	5 (20)	
Absolute lymphocyte count (cells/μl)		
Before treatment: median	1,670	
After concomitant RX/TMZ: median	920	*p* = 0.0002
Absolute neutrophil count (cells/μl)		
Before treatment: median	4,650	
After concomitant RX/TMZ: median	4,170	*p* = 0.14

### Flow Cytometry Analysis

Peripheral blood mononuclear cells (PBMC) were isolated using a lymphocyte separation medium (LSM, PAA) and frozen in 10% DMSO. They were stored in liquid nitrogen until analysis to allow analysis of all samples (time points) of a given patient in the same experiment. Samples were analyzed by flow cytometry directly *ex vivo* using several flow cytometry panels. The T/NK cell panel incorporated the CD45^PerCP−Cy5.5^, CD3^AA750^, CD4^ECD^, CD8^PE−Cy7^, CD16^FITC^, and CD56^PE^ antibodies (Supplementary Figure 2A). The monocyte/B cell panel incorporated the CD45^PerCP−Cy5.5^, CD14^PE^, and CD19^FITC^ antibodies (Supplementary Figure 2B). The granzyme B panel incorporated the CD3^ECD^, CD8^APC^ and granzyme B (+isotype)^PE−Cy5.5^ antibodies (Supplementary Figure 2C). The T cell naïve/memory panel incorporated the CD3^PE^, CD4^APC^, CD8^PE−Cy7^, CCR7^FITC^, and CD45RA^PerCP−Cy5.5^ antibodies (Supplementary Figure 2D). Treg phenotype was analyzed (i) *ex vivo* using an Fc block (Biolegend) and the CD3^FITC^, CD4^AA750^, CD25^PE−Cy7^, CD39^PE−CF594^, FoxP3 (+isotype)^A647^, Ki67^BV421^, and HLA-DR^PerCP−Cy5.5^ or PD1^BV421^ and CD45RA^PerCP−Cy5.5^ antibodies (Supplementary Figure 10A) and (ii) after 2 days of *in vitro* stimulation with CD3/CD28 beads at a bead to cell ratio of 1:1 (Invitrogen) using an Fc block and the CD4^AA750^, CD8^PE−Cy7^, PD1^BV421^, LAG3^FITC^, and ICOS^PE^ antibodies (Supplementary Figure 10B), with the corresponding isotype controls.

The CD3^AA750^, CD3^ECD^, CD4^ECD^, CD8^PE^, CD8^PE−Cy7^, CD8^APC^, CD14^PE^, CD16^FITC^, CD19^FITC^, CD25^PE−Cy7^, CD45^PerCP−Cy5.5^, CD45RA^PerCP−Cy5.5^, CD56^PE^, CCR7^FITC^, HLA-DR^PerCP−Cy5.5^, ICOS^PE^, and Ki67^BV421^ antibodies were obtained from BD Biosciences. The CD3^PE^, CD4^AA750^, CD39^PE−CF594^ antibodies were obtained from Beckman Coulter. The PD1^BV421^ antibody was obtained from Biolegend. The FoxP3 (+isotype)^A647^ and LAG3^FITC^ antibodies were obtained from ebioscience and the granzyme B (+isotype)^PE−Cy5.5^ antibodies were obtained from Invitrogen. The Live/Dead Yellow dye (Invitrogen) was used in all stainings to exclude dead cells. All surface stainings were performed at 4°C during 10 min in PBS containing 0.5% BSA and 0.05% sodium azide (Sigma). Staining with FoxP3 antibody was performed according to the manufacturer's instructions. Granzyme B staining was performed after cell fixation with 1% formaldehyde and permeabilization with 0.5% saponin. Cells were analyzed using a Gallios flow cytometer and Kaluza software (Beckman Coulter). The gating strategy and marker definition of subpopulations are depicted in Supplementary Figures 2, 10.

Cell proliferation was assessed in triplicate using 5(6)-carboxyfluorescein diacetate succinimidyl ester (CFSE, 0.6 μM, Sigma) 5 days after stimulation with CD3/CD28 beads (1/100, Invitrogen), SEB (20 ng/ml, Sigma), or medium (to assess background proliferation) (Supplementary Figure 2E).

Cell subsets were defined as: T cells: CD45^+^CD3^+^, CD4 T cells: CD45^+^CD3^+^CD4^+^, CD8 T cells: CD45^+^CD3^+^CD8^+^, B cells: CD45^+^CD19^+^, monocytes: CD45^+^CD14^+^, CD16^+^ cytotoxic NK cells: CD45^+^CD3^−^CD16^+^, and CD56^high^ cytokine-secreting NK cells: CD45^+^CD3^−^ CD56^high^ (Supplementary Figure 2). The percentage of granzyme B^+^ cells was calculated among CD3^+^CD8^+^ T cells. An isotype control to granzyme B was used to set the gating. T regulatory cells were defined as CD3^+^CD4^+^CD25^+^FoxP3^+^. An isotype control to FoxP3 was used to set the gating (Supplementary Figure 10). Naïve/memory cell subsets are defined as naïve (T_N_): CD45RA^+^CCR7^+^, central memory (T_CM_): CD45RA^−^CCR7^+^, effector memory (T_EM_): CD45RA^−^CCR7^−^, and effector (T_E_): CD45RA^+^CCR7^−^(Supplementary Figure 2D). Proliferation was analyzed using a stimulation index (SI) calculated as the ratio of the percentage of CD4 (or CD8)^+^CFSE^low^ cells among CD4 (or CD8) T cells in the presence of a stimulus (CD3/CD28 or SEB) relative to that in the presence of medium. This calculation is detailed in Supplementary Figure 2E.

Leukocyte, lymphocyte and neutrophil counts were obtained through complete blood count numeration performed during medical care at the corresponding time point. Counts for CD3, CD4, CD8 T, NK, and B cells were calculated by multiplying the percentage of the respective population obtained by flow cytometry by the lymphocyte count; counts for monocytes were calculated by multiplying the percentage of monocytes obtained by flow cytometry by the leukocyte count.

### Statistical Analysis

Data were analyzed using GraphPad Prism 7.02. Values for all patients or donors for a given parameter (cell counts, percentages of marker-expressing cells, stimulation indexes) were plotted using box and whiskers plots (showing median and 25th to 75th percentiles). Outliers were determined using the Tukey method [defined as >75th percentile + 1.5 interquartile range (IQR) or <25th percentile −1.5 IQR]. Cell variation over time was analyzed using a non-parametric Wilcoxon signed rank test. Unpaired *t*-test were used to compare age between patients and controls. Log-rank test was used to compare OS and PFS in patients with NLR > or <4. Fisher's exact test was used to compare sex distribution between patients and controls. Survival curves were generated using the Kaplan–Meier method to estimate OS and PFS rates. Spearman correlation was used to test correlation between PFS and lymphocyte count variation over time.

## Results

In order to assess impact of standard treatment on immune cell populations in patients with glioma, PBMC isolated from 24 patients with GBM and one patient with grade III astrocytoma were analyzed longitudinally over a minimum of 5 months (Figure 1), with follow-up samples every 3 months for a fraction of patients. Thirteen healthy controls (patients' spouses) were included in order to assess natural variation of the immune subsets.

Patients' characteristics are provided in Table 1. Patients and controls were similar in age (student *t*-test, *p* = 0.53) and sex (fisher's exact test, *p* = 0.31). Seven patients (28%) and 4 patients (16%) had a methylated and unmethylated MGMT promoter, respectively, whereas the methylation status was unknown for the remaining 13 patients (56%). IDH1/2 mutational status was known for six patients only (5 wt, 1 IDH1 mutated). All patients underwent 42-day TMZ administration concomitant to radiotherapy (Figure 1 and Table 1). Subsequently, all but one patient underwent cycles of adjuvant TMZ until progression or intolerability (mean number of cycles: 5.5, range 0–12). Total dose of TMZ received was variable (mean 13,265 mg, range: 5,760–18,550 mg). Radiotherapy doses were 59.4–60 Gy for 24 patients, with one patient benefitting from a boost to 72 Gy. Peripheral blood was taken before and along TMZ administration as depicted in Figure 1 for immune cell analysis.

**Figure 2:**
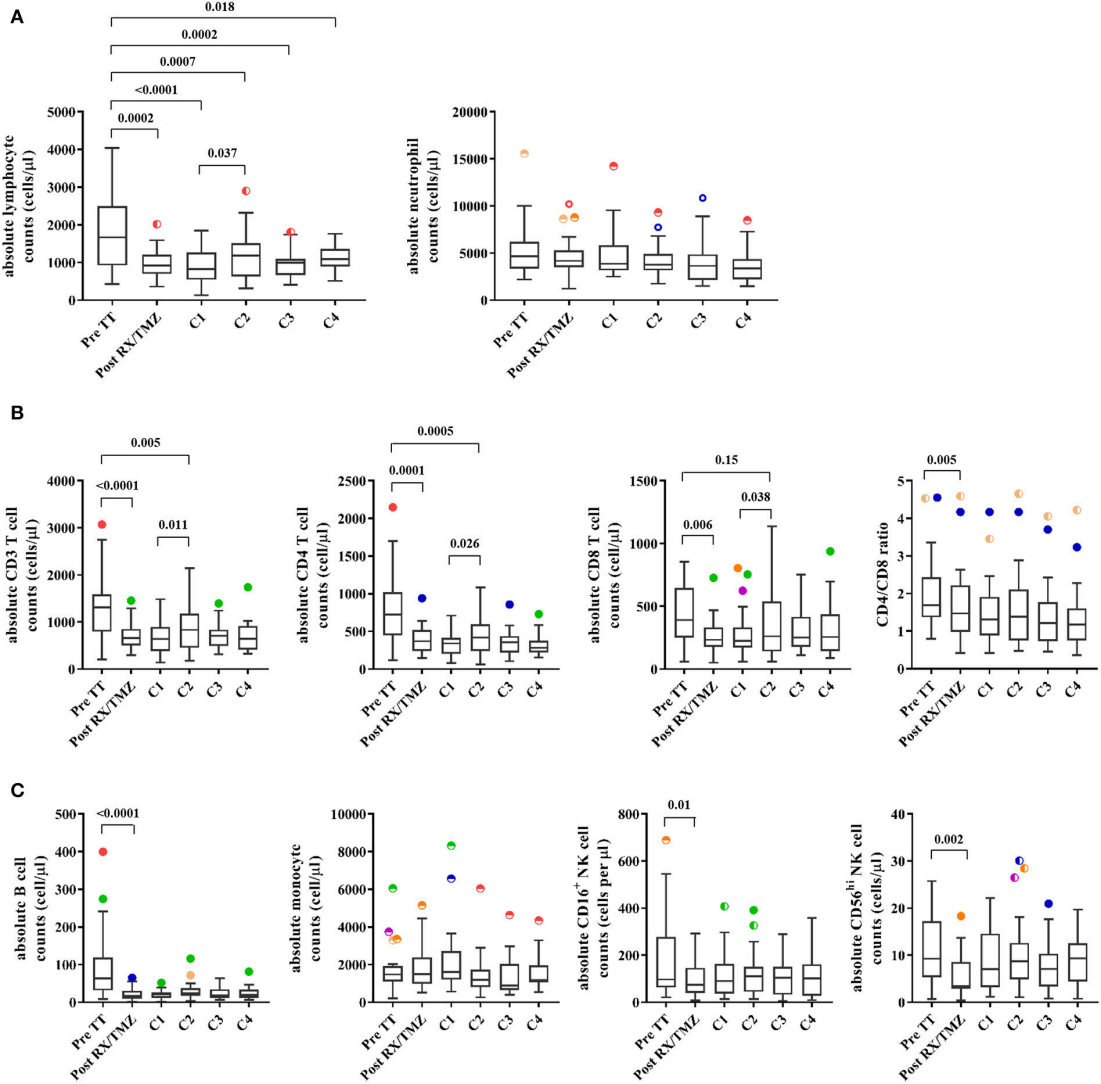
**(A)** The absolute lymphocyte counts (left panel) and neutrophil counts (right panel) are shown for patients (*n* = 25) before and upon treatment. **(B)** Absolute CD3, CD4, and CD8 T cell counts and CD4/CD8 ratio are shown for patients (*n* = 25) before and upon treatment. **(C)** Absolute B cell, monocyte, and CD16^+^ or CD56^high^ NK cell counts are shown for patients (*n* = 25) before and upon treatment. Wilcoxon signed rank test was used to test variation in absolute counts over time.

As expected, we observed that absolute lymphocyte counts were significantly reduced after concomitant radiochemotherapy (RX/TMZ, Figure 2A and Supplementary Figure 1A) and remained low during adjuvant TMZ treatment. Lymphopenia was not as severe as previously described (21), as only 24% of patients suffered grade 2 or more lymphopenia during the course of the study (Supplementary Figure 1B) and only one patient experienced grade 4 lymphopenia at only one time point during the study. In addition, although it is accepted that GBM induces lymphopenia by itself, 72% of patients had normal lymphocyte counts before introduction of concomitant RX/TMZ therapy. No patient was neutropenic before RX/TMZ administration and neutropenia was not observed thereafter (Figure 2A and Supplementary Figure 1A). It was previously shown that pre-treatment neutrophil-to-lymphocyte ratio (NLR)>4 was associated with poor prognosis in patients with glioma (33). Here, 20% of the patients had a NLR>4 before concomitant radiochemotherapy; however, this was not associated with a shorter OS (log-rank test, *p* = 0.10), nor PFS (log-rank test, *p* = 0.21). Variation in lymphocyte counts did not correlate with TMZ nor radiotherapy dose received. Recovery of lymphocyte counts was analyzed in a subset of patients for which blood counts were available long term after cessation of TMZ use and before introduction of a new course of chemotherapy (TMZ, lomustine, or irinotecan). Whereas lymphocyte counts 3 months post-TMZ discontinuation were still reduced as compared to pre-treatment counts, they reached pre-treatment numbers after 6 months, suggesting that there is no long-term effect of TMZ (Supplementary Figure 1C).

### Radiotherapy and TMZ Administration Induce Depletion of Several Cell Subsets

**Figure 3:**
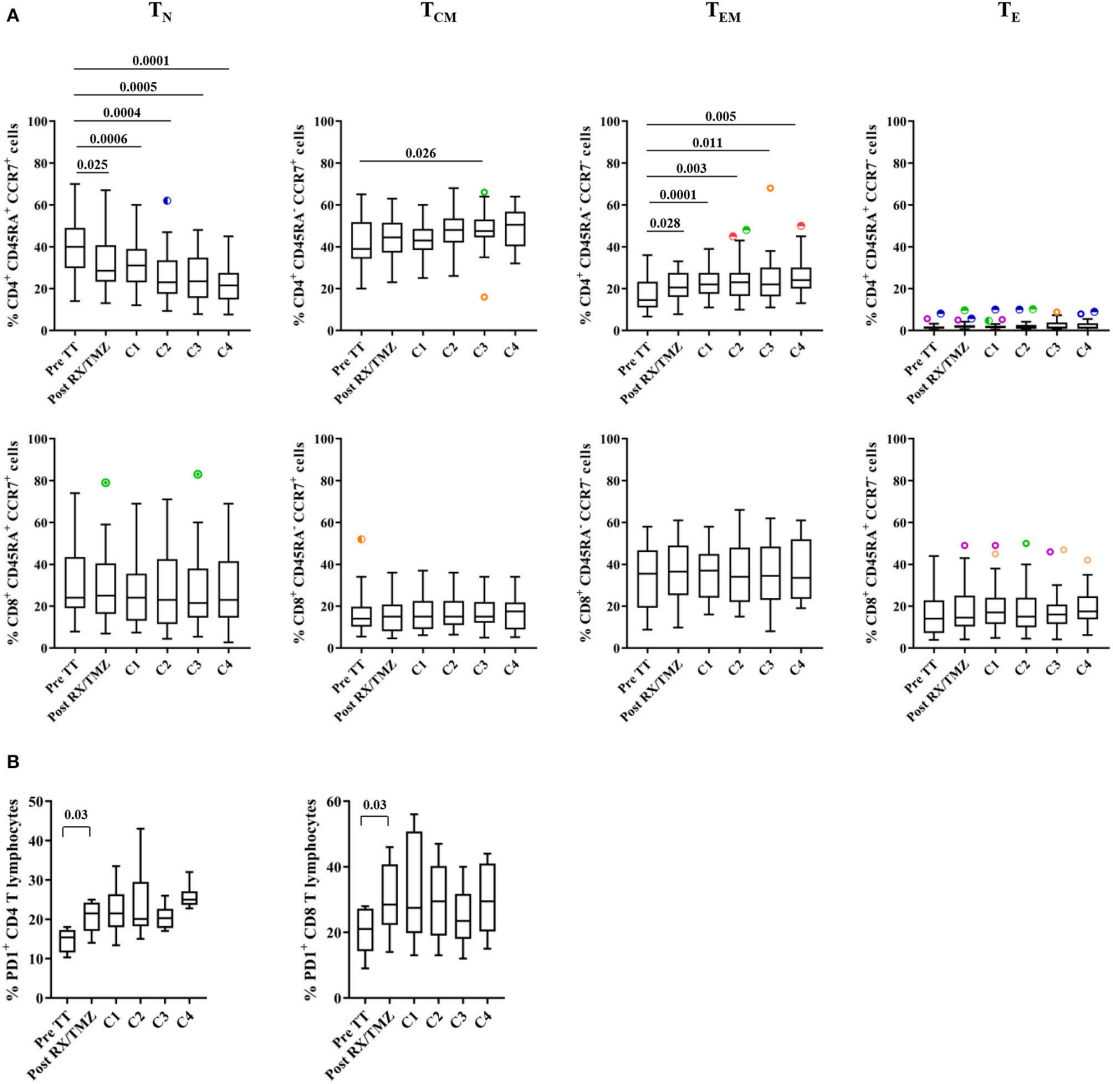
**(A)** The percentage of naïve (T_N_), central memory (T_CM_), effector memory (T_EM_), and effector cells (T_E_) in CD4 (upper panels) and CD8 (lower panels) are shown for patients (*n* = 25) before and upon treatment. **(B)** The percentages of CD4 (upper panel) and CD8 (lower panel) T cells expressing PD1 are shown for patients (*n* = 6) before and upon treatment. Data were analyzed using box and whiskers plots with outliers. Wilcoxon signed rank test was used to test variation in absolute counts over time.

We then investigated composition of the lymphocyte compartment in patients with glioma and healthy controls (exemplary stainings in Supplementary Figure 2). There was no significant difference in the percentage of cell subsets between patients and healthy controls before RX/TMZ initiation (Supplementary Figures 3A,B). Reduction in the percentage of CD4 T cells, B cells and of the CD4/CD8 ratio and increase in the percentage of monocytes was observed in patients compared with controls at various time points along treatment. Moreover, paralleling what we observed for absolute lymphocytes counts, RX/TMZ administration induced a strong and significant reduction in CD3, CD4, and CD8 T cell counts in patients (Figure 2B and Supplementary Figures 4A, 5A). Regarding CD3 and CD4 T cell counts, significant reduction was seen at all time points post-RX/TMZ initiation, with however a significant increase from TMZ cycle 1 to cycle 2, probably due to a general increase in lymphocyte counts at that time (Figures 2A,B). For CD8 T cell counts, this increase was observed as well, leading to restoration of cell counts as compared to pre-TT at the time of TMZ cycle 2 (Figure 2B). It has to be kept in mind that some of these parameters are interdependent. CD3, CD4, CD8, and B cell counts were correlated, probably due to introduction of the same factor in the count calculation (absolute counts for these populations were calculated using the absolute lymphocyte counts). In addition, percentages of CD4, CD8, and B cells correlated with the percentage of CD3 cells, but other parameters did not correlate with one another. The CD4/CD8 ratio was also significantly reduced. Absolute B cell, CD16^+^ cytotoxic, and CD56^hi^ cytokine-producing NK cell counts were similarly decreased after RX/TMZ administration, whereas monocytes were not modulated (Figure 2C and Supplementary Figures 4B, 5B). Reduction of cell subsets was persistent over adjuvant TMZ administration. Variation in the different immune cell counts did not correlate with TMZ dose (not shown). For 15 patients, immune subset analysis was available after cessation of TMZ. As shown in Supplementary Figure 3C, only B cell counts were restored 150–200 days after TMZ discontinuation. We next investigated whether the naïve/memory status of the CD4 and CD8 T cell subsets was modified over time. Indeed, whereas the percentage of naïve (T_N_, CD45RA^+^CCR7^+^), central memory (T_CM_, CD45RA^−^CCR7^+^), effector memory (T_EM_, CD45RA^−^CCR7^−^), or effector (T_E_, CD45RA^+^CCR7^−^) CD8 T cells did not vary over time, naïve CD4 T cells were significantly decreased, paralleled by an increase in effector memory cells (Figure 3A and Supplementary Figures 5C, 6A). This phenomenon was not observed in healthy individuals (Supplementary Figure 7A) and was not restored upon TMZ discontinuation (not shown). We also observed increased expression of PD1 on CD4 T cells upon concomitant RX/TMZ administration, which was also observed for CD8 T cells (Figure 3B and Supplementary Figure 6B) but not for healthy controls (Supplementary Figure 7B). PD1 expression was restricted to CD45RA^−^ cells.

### Antigen-Specific T Cell Proliferation Is Reduced After Concomitant RX/TMZ Administration

**Figure 4:**
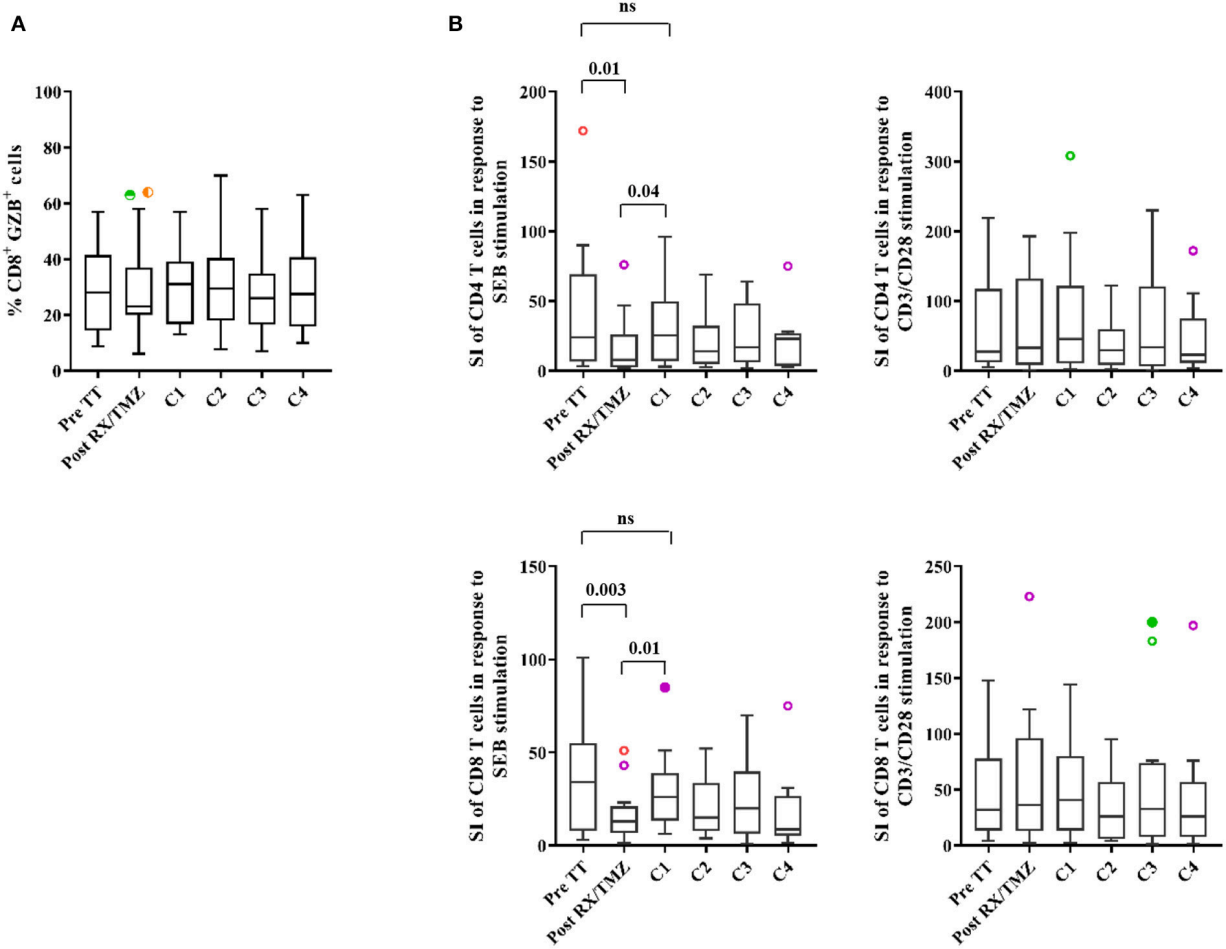
**(A)** The percentage of granzyme B (GZB)^+^ cells among CD8 T cells is shown for patients (*n* = 25) before and upon treatment. **(B)** Proliferation of CD4 (upper panels) and CD8 (lower panels) T cells in response to SEB (panels to the left) or CD3/CD28 antibodies (panels to the right) is shown for patients (*n* = 18) before and upon treatment. Data were analyzed using box and whiskers plots with outliers. Wilcoxon signed rank test was used to test variation in absolute counts over time.

We then assessed functional characteristics of CD4 and CD8 T cells along RX/TMZ administration. CD8 T cells containing preformed granzyme B (GZB) were detected but their percentage did not vary upon RX/TMZ administration (Figure 4A and Supplementary Figure 8A). CD4 and CD8 T cell proliferation upon stimulation with SEB, used as a physiological stimulus, or with CD3/CD28 beads was assessed for 18 patients and 9 controls for which enough PBMC were available. We observed a significant reduction in both CD4 and CD8 T cell proliferation upon stimulation with SEB after concomitant RX/TMZ treatment (Figure 4B, left panels and Supplementary Figure 8B). Reduction was transient and proliferation was restored at the time of the first cycle of adjuvant TMZ administration. Reduced proliferation upon concomitant RX/TMZ treatment was not observed upon incubation with a strong stimulus such as CD3/CD28 antibodies (Figure 4B, right panels) nor in controls (Supplementary Figure 9). Neither granzyme B expression by CD8 T cells nor CD4 and CD8 T cell proliferation correlated with the absolute counts or percentages of CD3, CD4, CD8, and Treg cells (not shown).

### The Percentage of Treg Cells Is Increased After Concomitant RX/TMZ Administration

**Figure 5:**
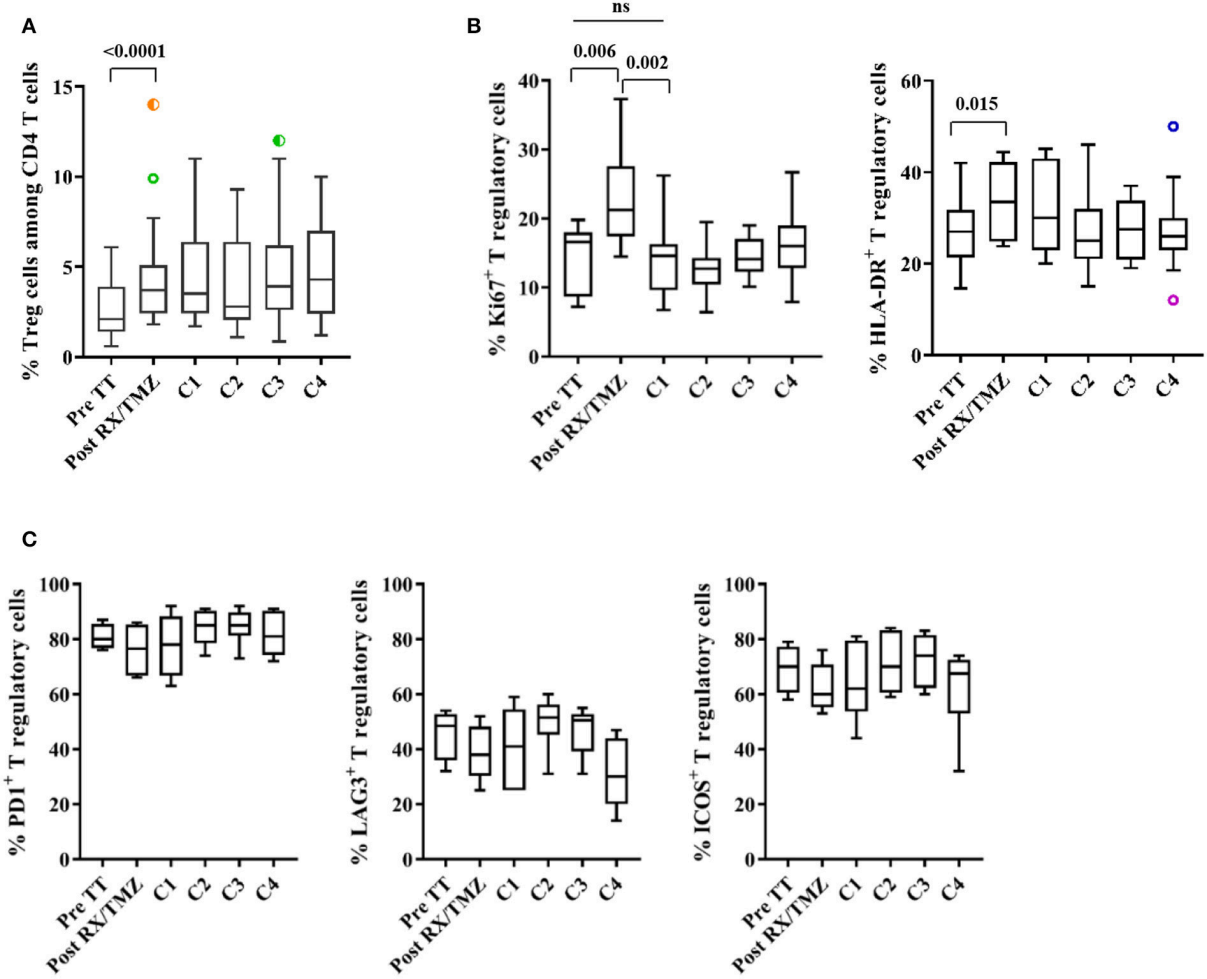
**(A)** The percentage of Treg (CD3^+^CD4^+^CD25^+^FoxP3^+^) cells among CD4 T cells is shown for patients (*n* = 25) before and upon treatment. **(B)** The percentage of Ki67^+^ (left) and of HLA-DR^+^ (right) Treg cells is shown for patients (*n* = 12) before and upon treatment. **(C)** The percentage of PD1^+^, LAG3^+^, and ICOS^+^ Treg cells is shown for patients (*n* = 6) over time. Data were analyzed using box and whiskers plots with outliers. Wilcoxon signed rank test was used to test variation in absolute counts over time.

The percentage of Treg cells (CD4^+^CD25^+^FoxP3^+^) among CD4 T cells was next investigated (see Supplementary Figure 10 for representative stainings). We observed a significant increase in Treg cells after concomitant RX/TMZ administration (Figure 5A and Supplementary Figure 11A). This was not observed in controls (Supplementary Figure 12A) and was not restored after TMZ discontinuation. The majority (>90%) of Treg cells were activated CD45RA^−^ Tregs, similarly to healthy controls, and this percentage did not vary over treatment administration (Supplementary Figure 12B). The percentage of Ki67^+^ proliferating Treg cells was transiently and significantly increased after concomitant RX/TMZ administration, and was accompanied by a significant increase in HLA-DR^+^ Treg cells (Figure 5B and Supplementary Figure 11B). These were not observed for controls nor in the total CD4 population (Supplementary Figure 12C). The percentage of CD39^+^ Treg cells was highly variable among patients and did not vary over time (Supplementary Figure 12D).

We were then interested in detecting expression of markers of activation/exhaustion, such as PD1, LAG3 and ICOS on Treg cells. As unambiguous detection of these markers was not seen *ex vivo*, we analyzed expression of PD1, LAG3, and ICOS after 48 h of *in vitro* stimulation with anti-CD3/anti-CD28 coated beads (Supplementary Figure 10B for representative stainings). The majority of Treg cells were able to express PD1 and ICOS after *in vitro* stimulation (Figure 5C and Supplementary Figure 11C) whereas a lower percentage of Treg cells expressed LAG3, not differently to what was observed in healthy controls (Supplementary Figure 12E).

### Correlation With Clinical Parameters

**Figure 6:**
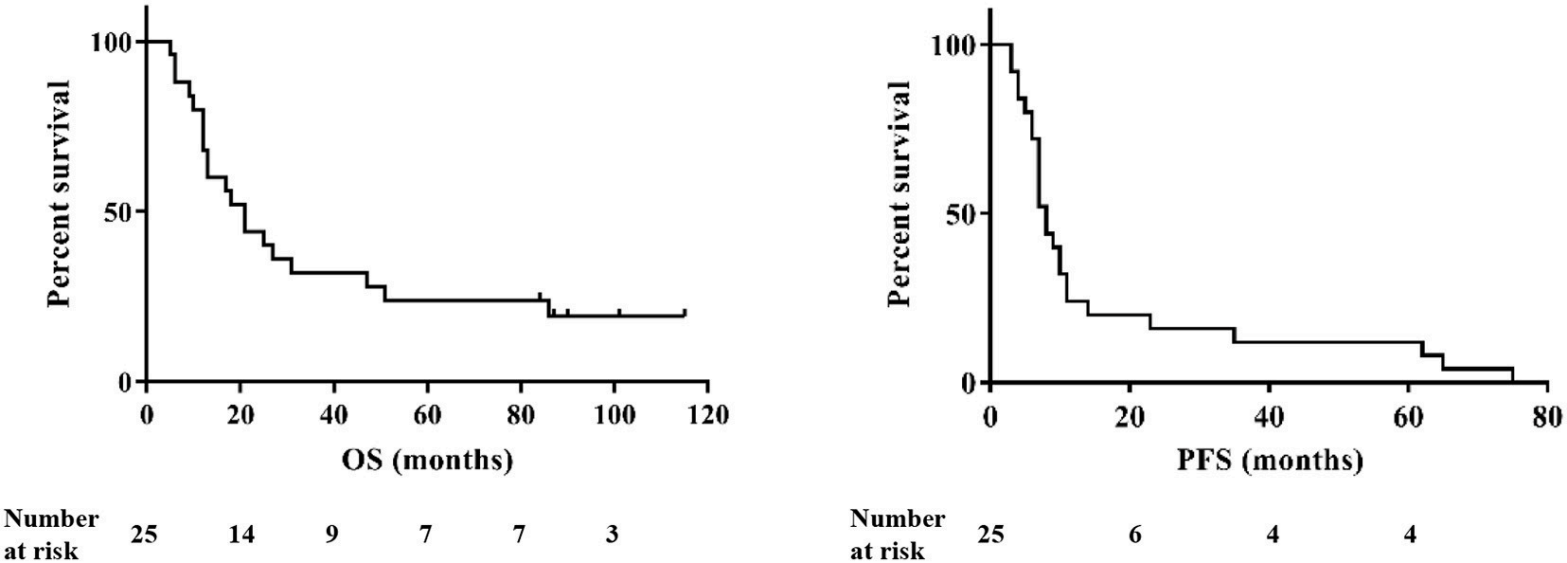
Overall survival (OS, left) and progression-free survival (PFS, right) of the patients (*n* = 25).

The median overall survival (OS) of the cohort was 21 months and the median progression-free survival (PFS) was 8 months (Figure 6). Six patients were long-term survivors (>60 months). They did not differ in absolute immune cell counts or variation over time from patients with shorter OS. OS and PFS did not correlate with age in our patient cohort.

## Discussion

Therapeutic vaccines and other immunotherapy strategies are being developed for patients with glioma, but efficacy has still to be improved for clinical benefit to be reached. The explanation for the lack of vaccine efficacy is multifactorial and includes suboptimal immunization, suboptimal homing of the elicited T cells to the brain, and impaired T cell function in the tumor microenvironment. Parameters influencing vaccine immunization efficacy encompass nature of the antigen, choice of the adjuvant(s), injection route and frequency, and patient immunosuppression. The latter is mainly the result of immunosuppressive treatments patients receive and it is therefore of importance to understand the impact of standard of care treatment on the peripheral immune system of patients with high-grade glioma. Studies assessing the impact of concomitant RX/TMZ treatment on immune cells have previously been published (34). Our study contributes to this field, bringing additional information from inclusion of a healthy control group, in which immune subpopulation analysis was performed, and assessment of T cell function.

We show here that concomitant RX/TMZ induces a strong reduction in lymphocyte counts, which is however reversible upon TMZ discontinuation. This is contrary to previous reports suggesting that lymphocytes are depleted for more than 1 year (35). In our study, a relatively high proportion of patients experienced 6 months or more without receiving TMZ or other chemotherapeutic drugs after initial treatment. We were therefore able to test restoration of lymphocyte counts in patients not receiving irinotecan, lomustine, or further TMZ. It is interesting to note that, in our cohort, bevacizumab did not induce further lymphopenia, as lymphocyte counts after TMZ discontinuation in patients receiving or not bevacizumab were not statistically different (not shown). This might favor vaccination in recurrent glioma patients, and we are currently testing this in a clinical trial of multipeptide vaccination with or without pembrolizumab in patients with relapsing GBM (NCT03665545). Studies have suggested that the main factor for lymphopenia might be radiotherapy (28). In our study, adjuvant cycles of TMZ did not worsen lymphopenia, which goes into this direction. Pre-treatment and treatment-related lymphopenia did not correlate with OS, as previously suggested (21). A limitation from our study lies in the fact that steroid doses were not prospectively collected, preventing assessment of its role into account.

Analysis of the control group showed that immune cell subset composition does not significantly vary over time. The limitation regarding these data comes from the fact that blood counts were not available for controls, preventing calculation of absolute cell counts. In patients, drop in CD4 T cells was more pronounced than that of CD8 T cells, as evidenced by decrease in the CD4/CD8 T cell ratio, in accordance with previous studies (20, 22). This strong decrease in CD4 T cells was accompanied by reduction in the naïve subset. This observation is interesting in the light of the recent publication showing sequestration of naïve T cells in the bone marrow of patients with glioma (36). However, as the latter study was performed in treatment-naïve patients, whether this occurs similarly upon treatment still needs to be determined. Concomitant increase in memory CD4 T cells might also suggest naïve T cell activation and transition to a memory phenotype. This is supported by the increase in the percentage of PD1^+^ CD4 and CD8 T cells upon RX/TMZ treatment. T cell activation probably results from homeostatic proliferation to replenish the T cell pool depleted by radiochemotherapy (37). Alternatively, PD1^+^ T cells could be antigen-specific T cells generated through radio- or chemotherapy-induced immunogenic tumor cell death (38). Altogether, the fact that these variations are not observed in healthy individuals suggests a direct effect of radiochemotherapy and confirms the importance of including a control group in the analysis.

Our study provides for the first time insights into the effect of radiochemotherapy on T cell function in patients with glioma. We show that CD4 and CD8 T cell proliferation is transiently reduced upon RX/TMZ administration, when an antigenic stimulus is used. Restoration of T cell proliferation during adjuvant TMZ administration suggests that radiotherapy plays a significant role in proliferation modulation. Altogether, transient reduction in proliferation associated with strong lymphopenia during RX/TMZ treatment suggests that vaccination should be performed outside the concomitant radiochemotherapy administration window.

Finally, our observation that the proportion of Treg cells among CD4 T cells is increased during radiochemotherapy, and, as previously observed (35), is not restored on the long term, suggest that depleting Tregs might be required in future vaccination trials. In this regard, cyclophosphamide has been shown to reduce Treg numbers and allow elicitation of higher levels of T cells responses (39). In our study, Tregs were CD45RA^−^ effector cells, with potential suppressive activity (40). However, expression of PD1, ICOS and LAG-3, although addressed after *in vitro* stimulation, might suggest that these cells are not highly suppressive. Indeed, it has recently been shown that glioma-infiltrating Tregs expressed PD1, ICOS, and Tim-3, and that PD1-expressing Tregs were impaired in their suppressive activity (41). In this regard, induction of checkpoint molecules on Treg cells by radiochemotherapy would therefore be beneficial.

Altogether, results from the various studies assessing the impact of standard of care treatment, including the current one, should not prevent initiation of clinical trials of therapeutic vaccination in patients with high-grade glioma. In this regard, we showed in a previous study that patients with glioma vaccinated for seasonal influenza were able to elicit antibody responses (42), suggesting that induction of immune responses to viral antigens in lymphopenic patients is efficient. The timing of optimal administration of therapeutic glioma vaccination is still unknown. In this regard, a study comparing initiation of vaccination before or after concomitant RX/TMZ reported no differences in elicitation of antigen-specific CD8 T cell responses in the two groups of patients (43). However, although not experimentally validated, results of the current study suggest that, due to potentially suboptimal T cell elicitation, vaccination during concomitant radiochemotherapy should be avoided. In addition, strategies aiming at depleting Treg cells should be implemented in future trials.

## Data Availability Statement

The raw data supporting the conclusions of this article will be made available by the authors, without undue reservation, to any qualified researcher.

## Ethics Statement

This study was carried out in accordance with the recommendations of the Swiss Ethics Committees on research involving humans, with written informed consent from all subjects. All subjects gave written informed consent in accordance with the Declaration of Helsinki. This protocol was approved by the Ethics Committee of Geneva, Switzerland.

## Author Contributions

VD and P-YD designed the study. GP and VW performed the experiments. VD analyzed the data. EM performed statistical analysis. AV, DM, and KS provided patients and patient data. VD wrote the manuscript. VD, EM, AV, DM, KS, and P-YD revised the manuscript.

### Conflict of Interest

The authors declare that the research was conducted in the absence of any commercial or financial relationships that could be construed as a potential conflict of interest.
