# Supplementary material for: Impact of Radiochemotherapy on Immune Cell Subtypes in High-Grade Glioma Patients
Source: Front Oncol. 2020 Feb 14;10:89. doi: 10.3389/fonc.2020.00089 (PMC7034105; doi:10.3389/fonc.2020.00089)
Supplement: Supplementary file 1 [file Data_Sheet_1.PDF]

Supplementary Material

1.1 Supplementary Figures

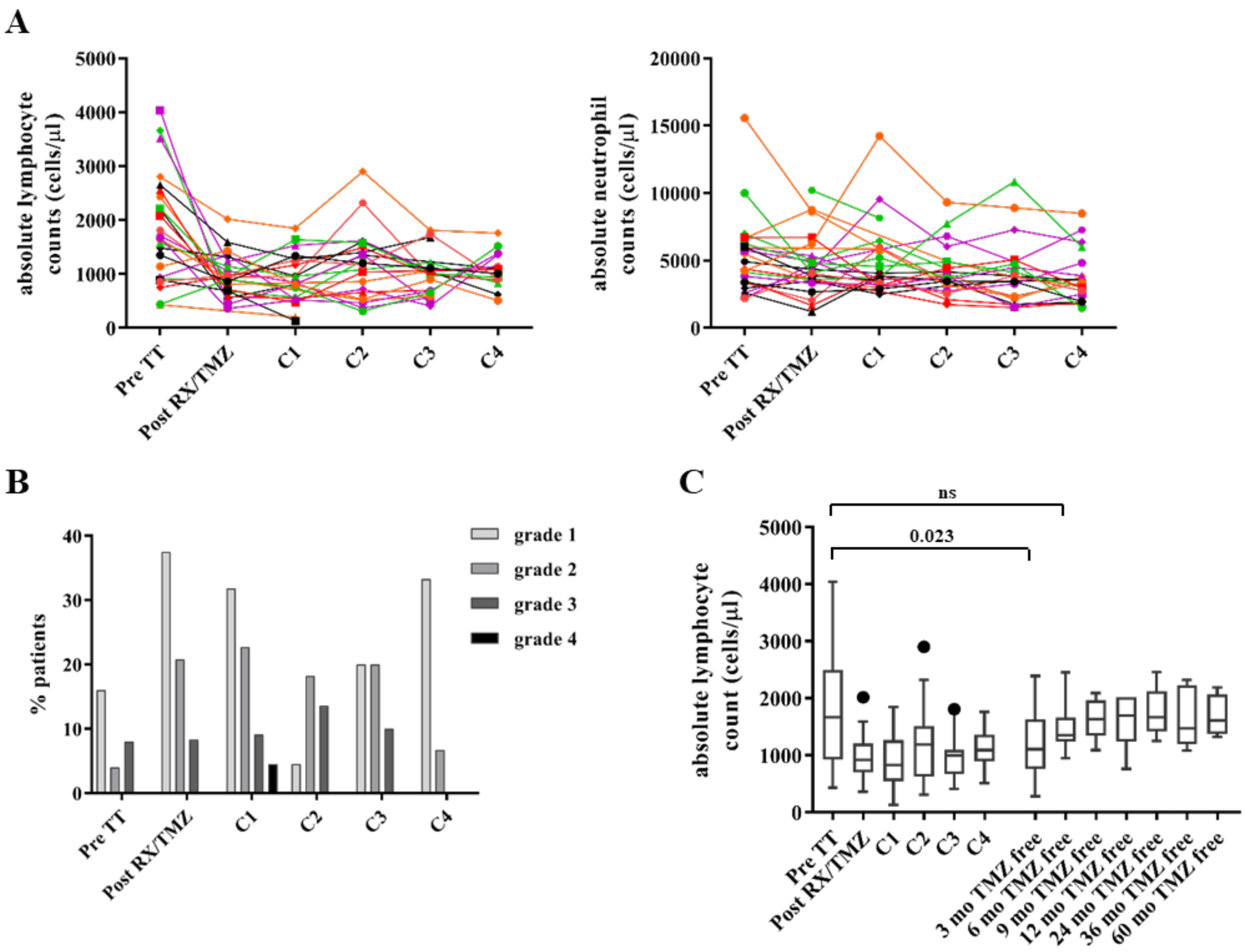

Supplementary Figure 1. (**A**) The absolute lymphocyte counts (left panel) and neutrophil counts (right panel) are shown for patients (n=25) before and upon treatment. (**B**) Percentage of patients with grade 1, 2, 3 or 4 lymphopenia before and upon treatment. Patients not suffering from lymphopenia are not shown on the graph. (**C**) Absolute lymphocyte counts are shown for patient 3 months (n=20), 6 months (n=13), 9 months (n=8), 12 months (n=7), 24 months (n=6), 36 months (n=5) and 60 months (n=4) after TMZ discontinuation.

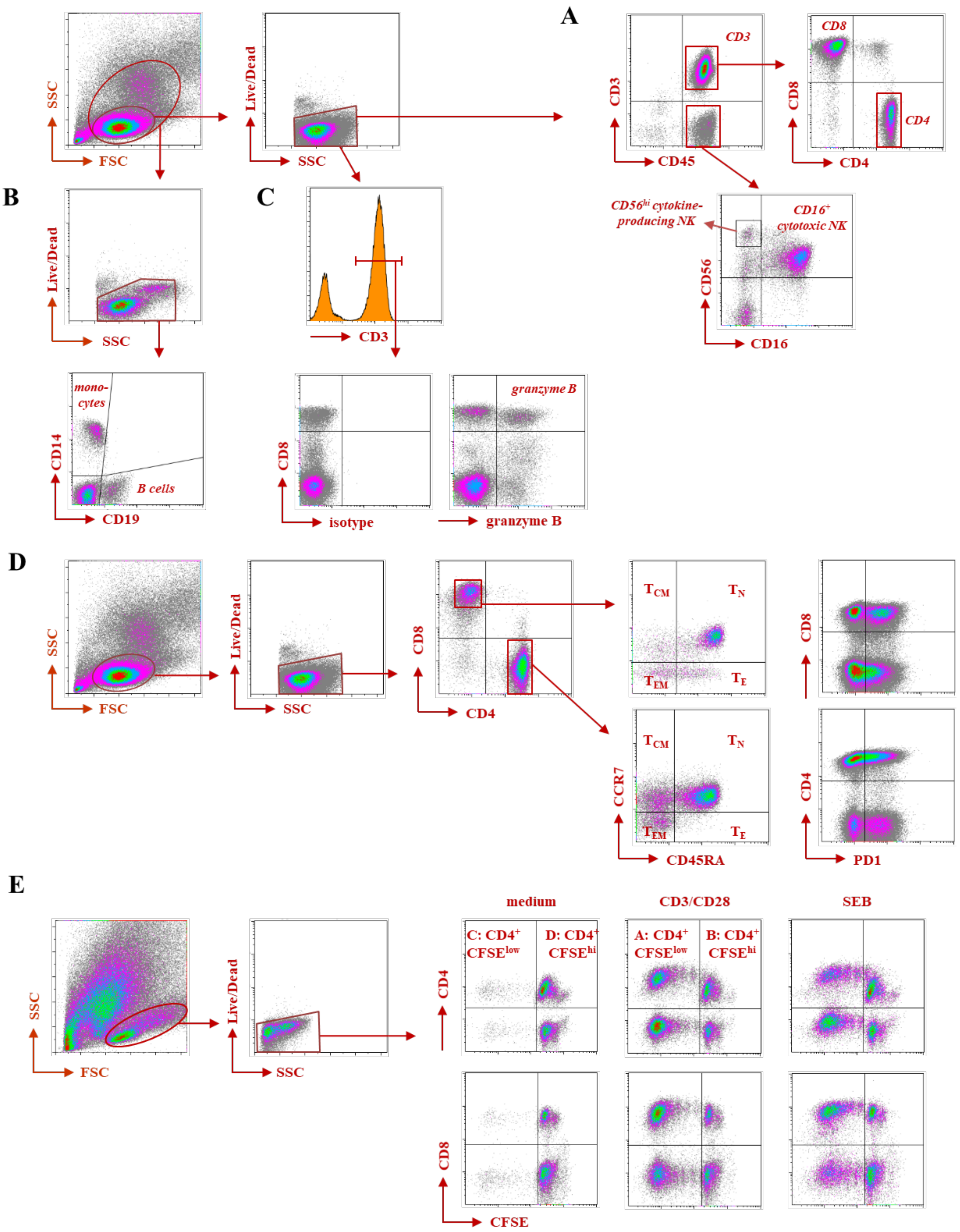

Formula used to calculate the stimulation index (SI): example for CD4 T cell proliferation after stimulation with CD3/CD28 beads:

$$SI_{CD3/CD28} = \text{\% divided CD4 cells in presence of CD3/CD28 beads} \,/\, \text{\% divided CD4 cells in presence of medium}$$

$$SI_{CD3/CD28} = \left(\frac{\%CD4^{+}CFSE^{low}\text{ cells (A)}}{\%\text{ total }CD4^{+}\text{ cells (A+B)}}\right) \Big/ \left(\frac{\%CD4^{+}CFSE^{low}\text{ cells (C)}}{\%\text{ total }CD4^{+}\text{ cells (C+D)}}\right)$$

Supplementary Figure 2. Strategy used for flow cytometry analysis of T and NK cell subsets (**A**), B cell and monocyte subsets (**B**), granzyme B expression by CD8 T cells (**C**), naïve/memory phenotype (**D**) and proliferation (**E**).

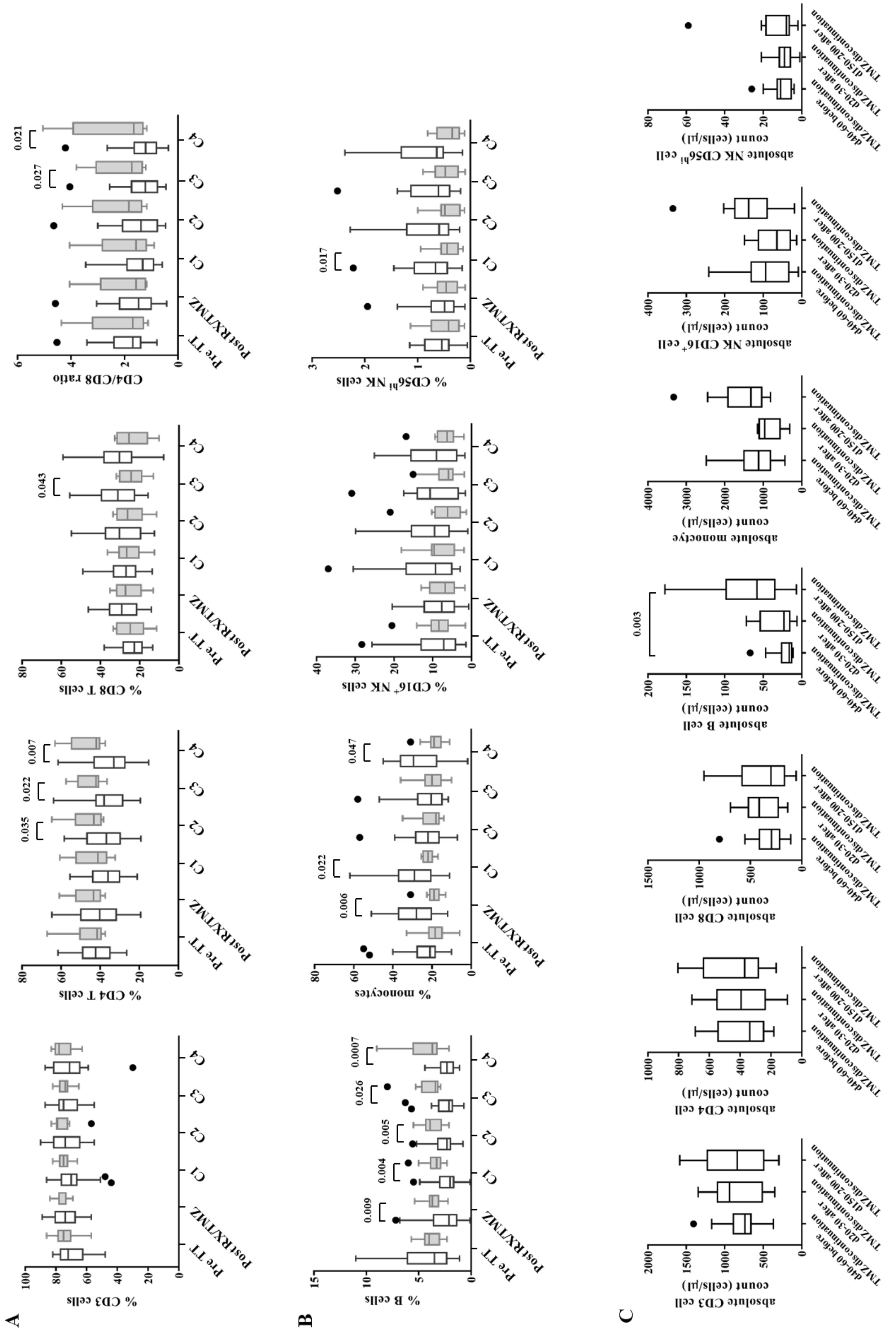
A
% CD3 cells
% CD4 T cells
% CD8 T cells
CD4/CD8 ratio
B
% B cells
% monocytes
% CD16+ NK cells
% CD56hi NK cells
C
absolute CD3 cell count (cells/µl)
absolute CD4 cell count (cells/µl)
absolute CD8 cell count (cells/µl)
absolute B cell count (cells/µl)
absolute monocyte count (cells/µl)
absolute NK CD16+ cell count (cells/µl)
absolute NK CD56hi cell count (cells/µl)
Pre TT
PostRX/TMZ
C1
C2
C3
C4
d40-60 before TMZ discontinuation
d20-30 after TMZ discontinuation
d150-200 after TMZ discontinuation

Supplementary Figure 3. (**A**) The percentages of CD3, CD4 and CD8 T cells and the CD4/CD8 ratio are shown for patients (open boxes, n=25) and for controls (grey boxes, n=13) before and upon treatment. (**B**) The percentages of B cells, monocyte and $CD16^{+}$ or $CD56^{high}$ NK cells are shown for patients (open boxes, n=25) and for controls (grey boxes, n=13) before and upon treatment. (**C**) Absolute CD3, CD4 and CD8 T cell, B cell, monocyte and $CD16^{+}$ or $CD56^{high}$ NK cell counts are shown for patients (n=15) 40-60 days before and 20-30 days and 150-200 days after TMZ discontinuation. Data were analyzed using box and whiskers plots with outliers. Wilcoxon signed rank test was used to test variation in percentages over time.

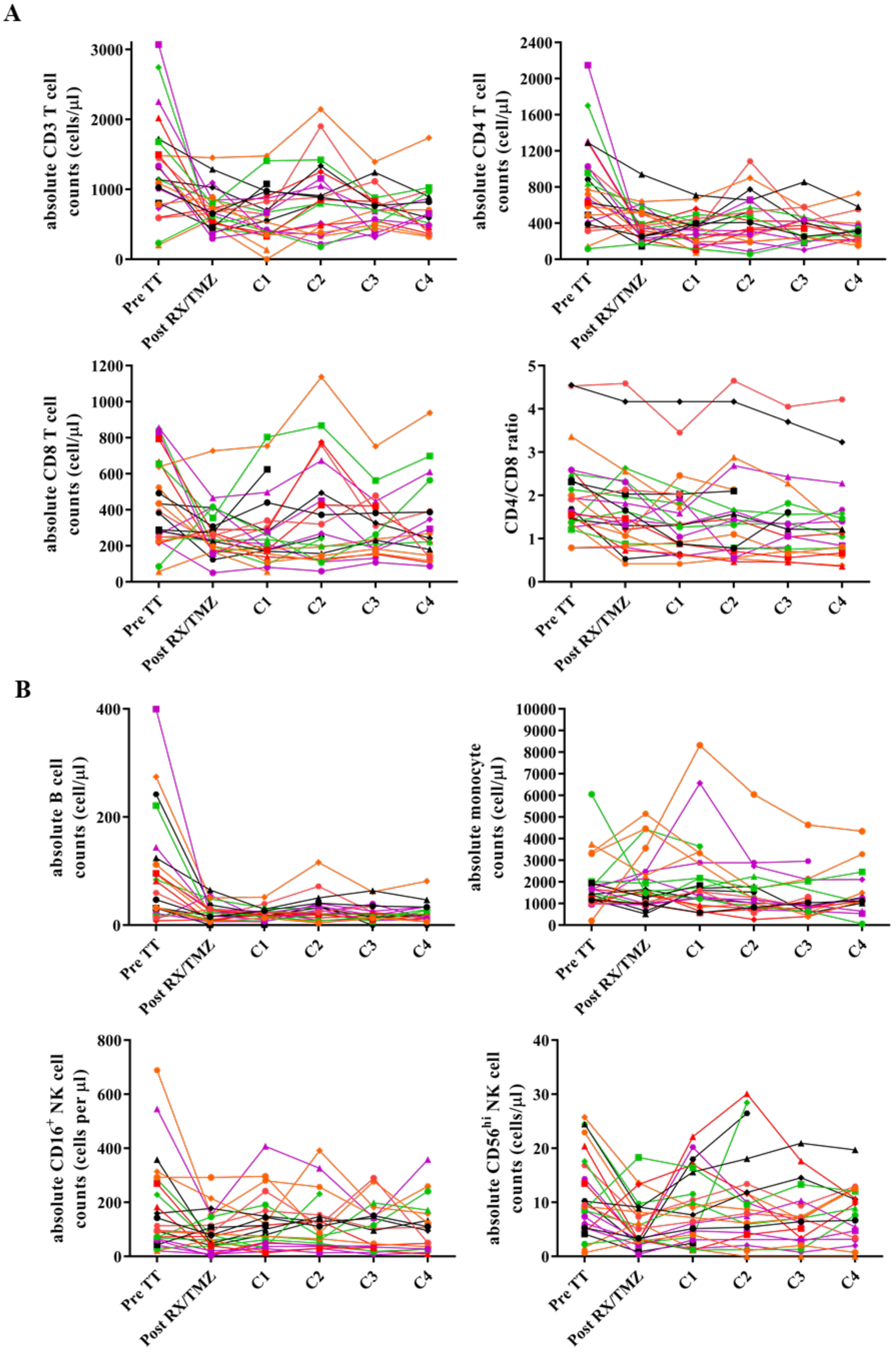

Supplementary Figure 4. (**A**) Absolute CD3, CD4 and CD8 T cell counts and CD4/CD8 ratio are shown for patients (n=25) before and upon treatment. (**B**) Absolute B cell, monocyte and $CD16^{+}$ or $CD56^{high}$ NK cell counts are shown for patients (n=25) before and upon treatment. A different symbol shape and color combination is given for each individual patient.

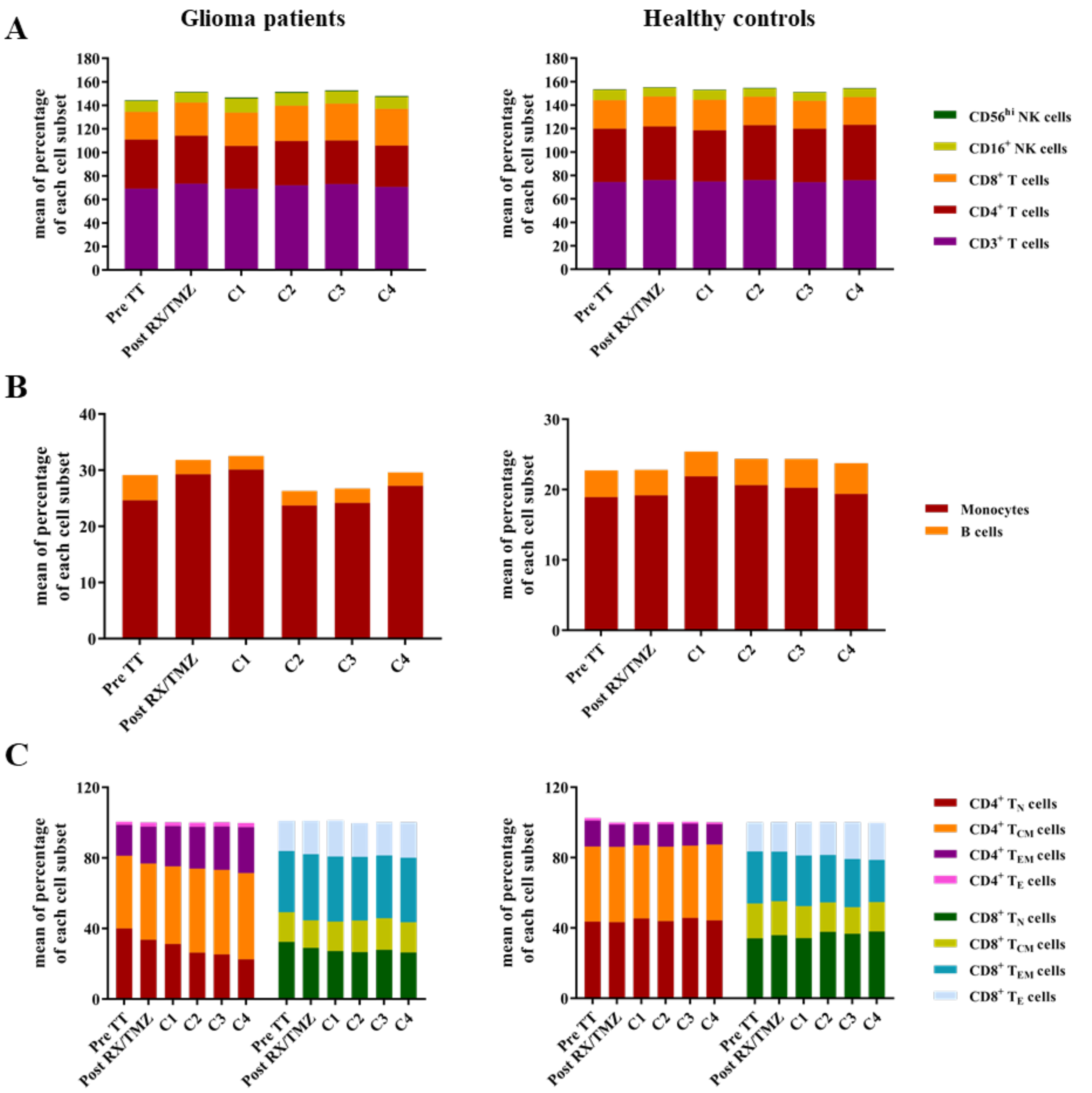

Supplementary Figure 5. Mean of the percentage of each subpopulation from a given flow cytometry analysis is given for patients (n=25) and healthy controls (n=13). (**A**) T and NK cell subsets, (**B**) B cell and monocyte subsets, (**C**) naïve/memory CD4 and CD8 T cell subsets, as defined in suppl. Fig. 2.

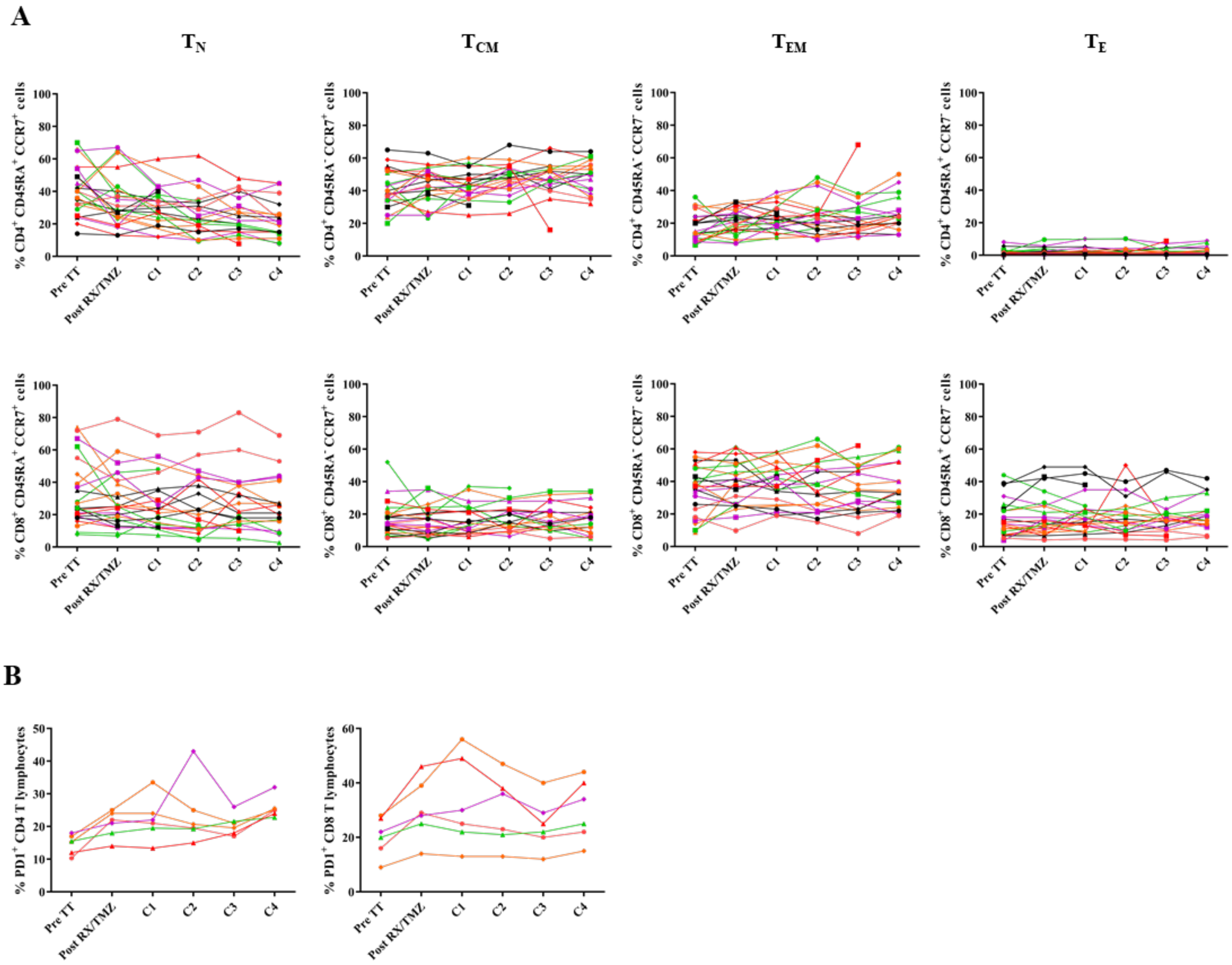

Supplementary Figure 6. (**A**) The percentage of naïve (T_N), central memory (T_{CM}), effector memory (T_{EM}) and effector cells (T_E) in CD4 (upper panels) and CD8 (lower panels) are shown for patients (n=25) before and upon treatment. (**B**) The percentages of CD4 and CD8 T cells expressing PD1 are shown for patients (n=6) before and upon treatment. A different symbol shape and color combination is given for each individual patient.

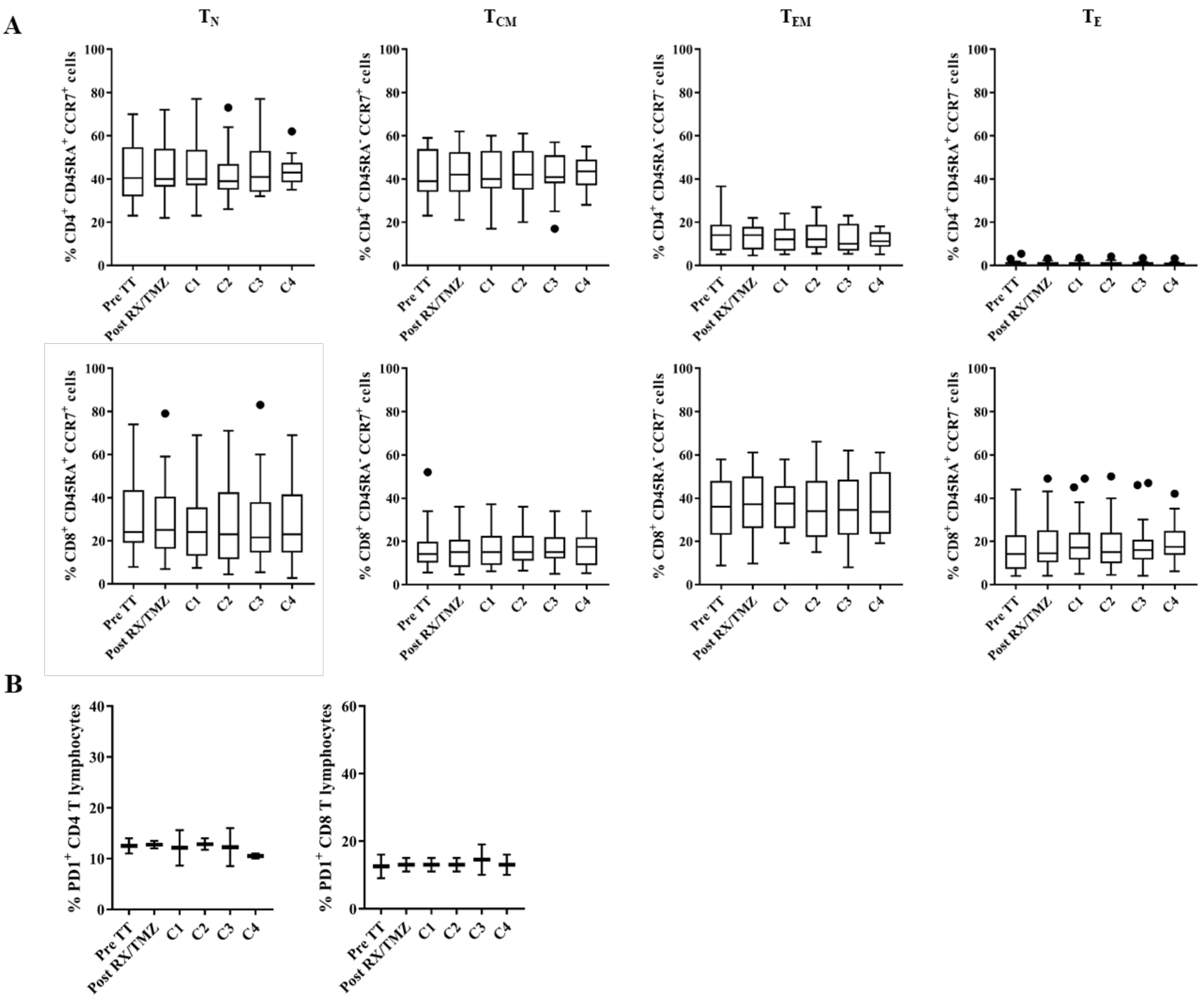

Supplementary Figure 7. (**A**) The percentage of native (T_N), central memory (T_{CM}), effector memory (T_{EM}) and effector cells (T_E) in CD4 (upper panels) and CD8 (lower panels) are shown for controls (n=13). (**B**) The percentages of CD4 (upper panel) and CD8 (lower panel) T cells expressing PD1 are shown for controls (n=3) before and upon treatment. Data were analyzed using box and whiskers plots with outliers. Wilcoxon signed rank test was used to test variation in percentages over time.

A

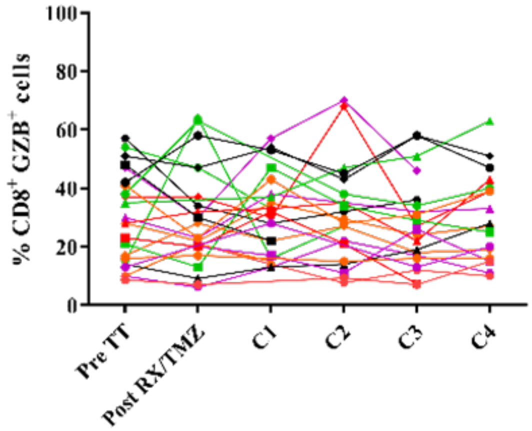

B

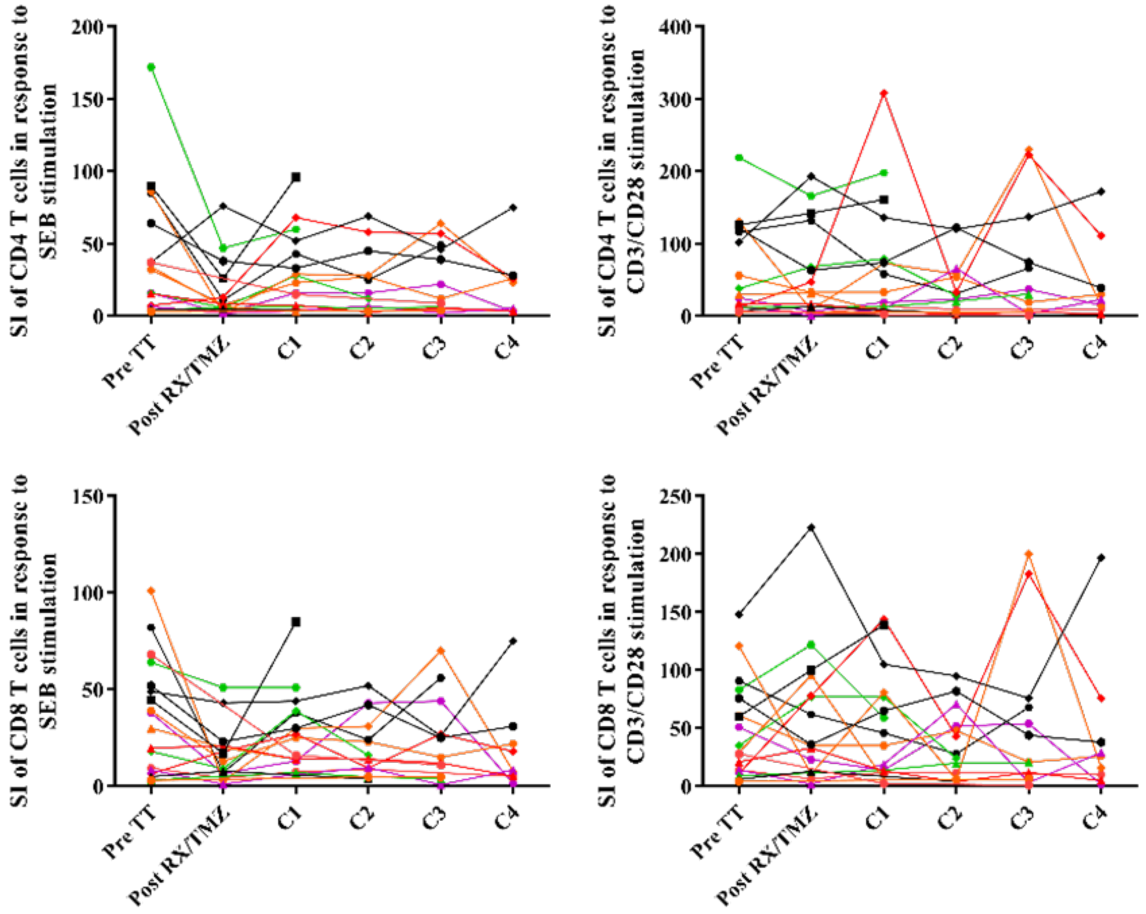

Supplementary Figure 8. (**A**) The percentage of granzyme B (GZB)$^+$ cells among CD8 T cells is shown for patients (n=25) before and upon treatment. (**B**) Proliferation of CD4 (upper panels) and CD8 (lower panels) T cells in response to SEB (panels to the left) or CD3/CD28 antibodies (panels to the right) is shown for patients (n=18) before and upon treatment. A different symbol shape and color combination is given for each individual patient.

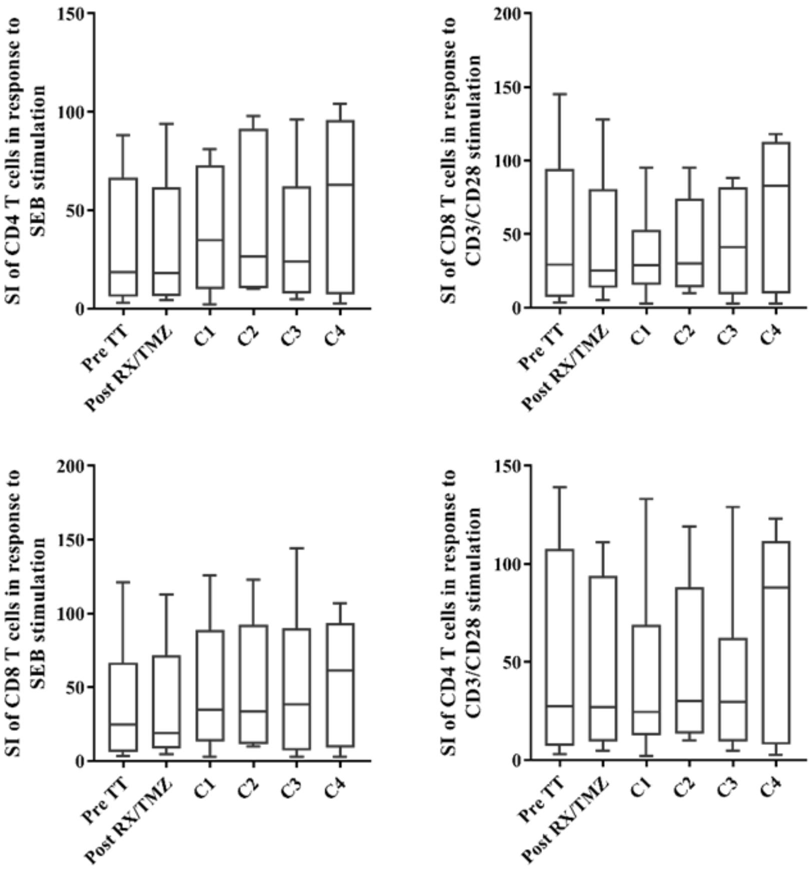

Supplementary Figure 9. Proliferation of CD4 (upper panels) and CD8 (lower panels) T cells in response to SEB (panels to the left) or CD3/CD28 antibodies (panels to the right) is shown for controls (n=9). Data were analyzed using box and whiskers plots with outliers. Wilcoxon signed rank test was used to test variation in percentages over time.

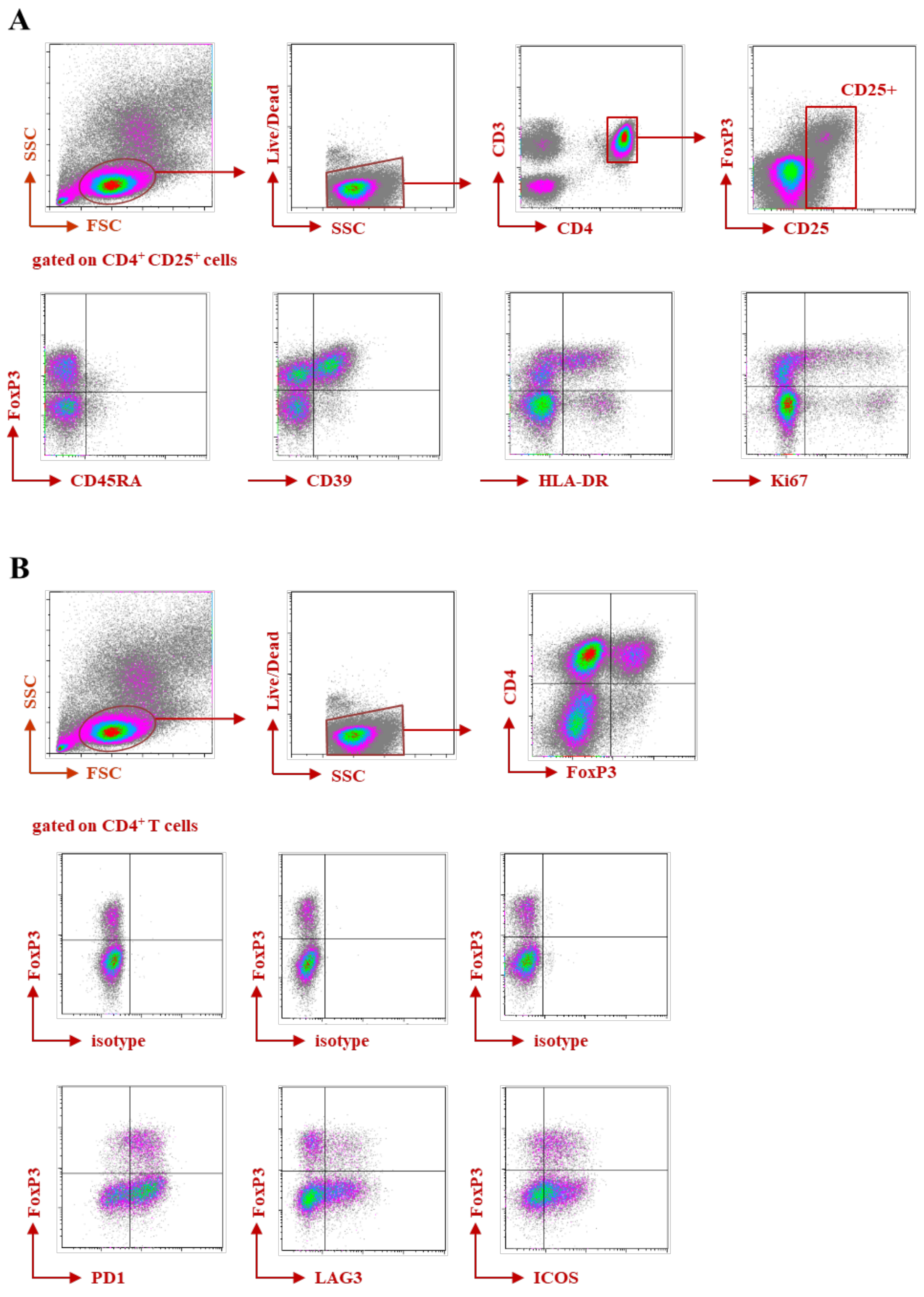

Supplementary Figure 10. Strategy used for flow cytometry analysis of Treg phenotype directly *ex vivo* (A) or after 48h of in vitro stimulation with CD3/CD28 beads (B).

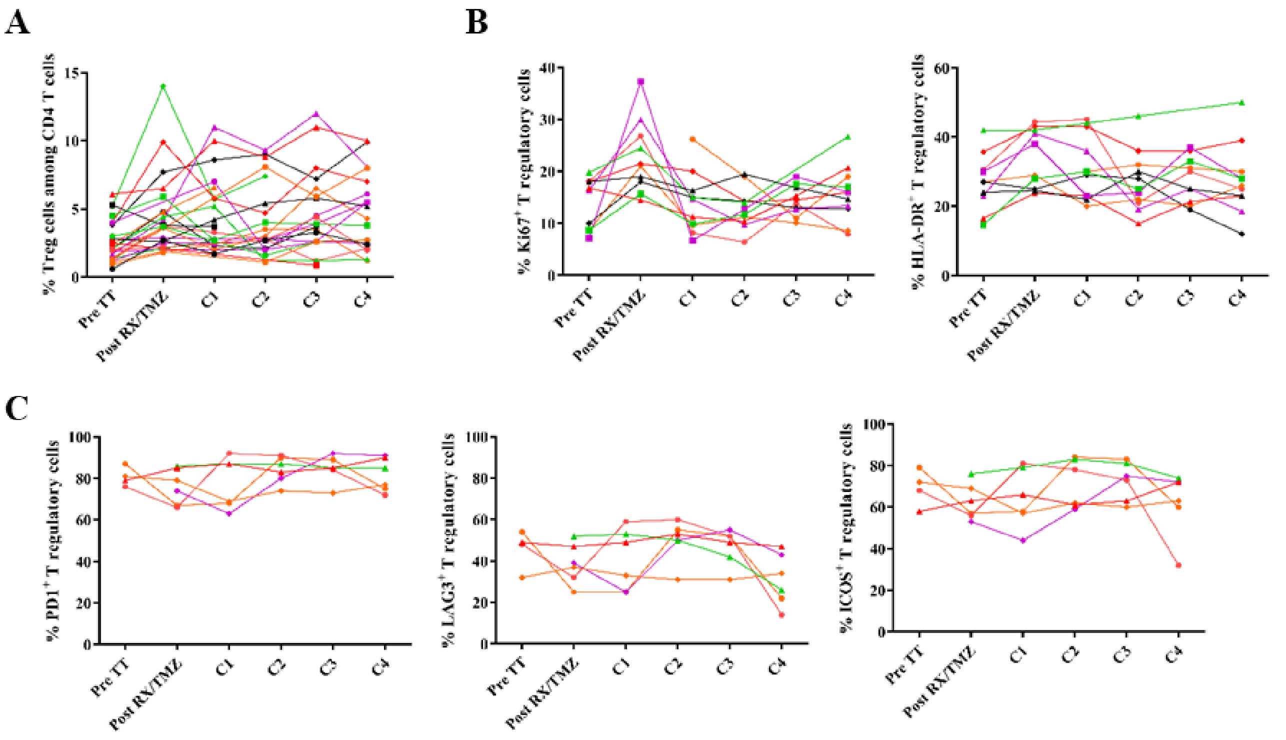

Supplementary Figure 11. (**A**) The percentage of Treg ($CD3^{+}CD4^{+}CD25^{+}FoxP3^{+}$) cells among CD4 T cells is shown for patients (n=25) before and upon treatment. (**B**) The percentage of $Ki67^{+}$ (left) and of $HLA\text{-}DR^{+}$ (right) Treg cells is shown for patients (n=12) before and upon treatment. (**C**) The percentage of $PD1^{+}$, $LAG3^{+}$ and $ICOS^{+}$ Treg cells is shown for patients (n=6) over time. A different symbol shape and color combination is given for each individual patient.

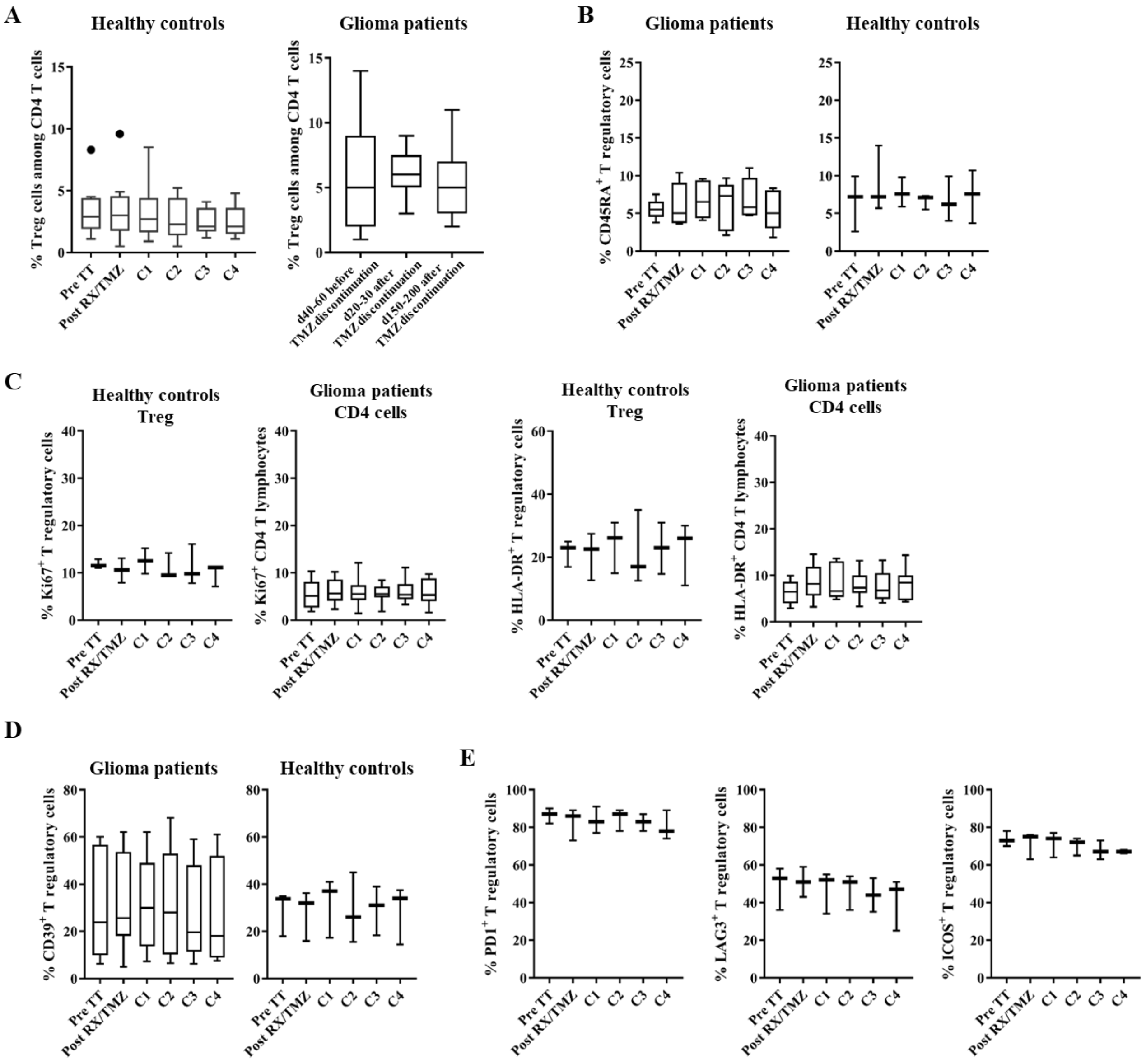

Supplementary Figure 12. (**A**) The percentage of Treg ($CD3^+CD4^+CD25^+FoxP3^+$) cells among CD4 T cells is shown for controls (n=13) along time (left panel) and percentage of Treg cells among CD4 T cells is shown for patients (n=15) 40-60 days before and 20-30 days and 150-200 days after TMZ discontinuation (right panel). (**B**) The percentage of $CD45RA^+$ Treg cells is shown for patients (left, n=12) and for controls (right, n=3). (**C**) The percentage of $Ki67^+$ (left panels) and $HLA\text{-}DR^+$ (right panels) cells is shown for controls among Treg cells (n=3) and for patients among total CD4 T cells (n=12). (**D**) The percentage of $CD39^+$ Treg cells is shown for patients (left, n=12) and for controls (right, n=3). (**E**) The percentage of $PD1^+$, $LAG3^+$ and $ICOS^+$ Treg cells is shown for controls (n=3). Data were analyzed using box and whiskers plots with outliers. Wilcoxon signed rank test was used to test variation in absolute counts over time.
